# Comments about the comparative bulk RNA sequencing between palmoplantar pustulosis and dyshidrotic palmoplantar eczema

**DOI:** 10.12688/f1000research.159565.1

**Published:** 2024-12-06

**Authors:** Kazuki Yatsuzuka, Jun Muto, Masamoto Murakami

**Affiliations:** 1 Department of Dermatology, Ehime University, Toon, Ehime Prefecture, 791-0295, Japan; 2 Department of Anatomy, Miyazaki University, Miyazaki, Miyazaki Prefecture, 889-1692, Japan

**Keywords:** palmoplantar pustulosis, dyshidrotic eczema, psoriasis, RNA sequencing, intraepidermal vesicle

## Abstract

This correspondence discusses the recent findings by Straalen et al., highlighting molecular similarities and distinctions between palmoplantar pustulosis (PPP) and dyshidrotic palmoplantar eczema (DPE). The study emphasizes shared proinflammatory pathways and T-cell–related gene upregulation while detailing unique features such as neutrophil involvement in PPP and lipid antigen processing in DPE. We elaborate on histopathological differences, especially intraepidermal vesicle formation in PPP linked to IL–1–mediated pathways and the absence of hyaluronan expression, contrasting with Th2 cytokines-driven spongiosis in DPE. By addressing IL-4, hyaluronan synthases, and keratinocyte adhesion molecules, this correspondence aims to deepen understanding of PPP and DPE pathophysiology.

To the Editor:

We read with great interest the recent report by Straalen et al. ^1^ describing molecular overlap among inflammatory palmoplantar diseases, including palmoplantar pustulosis (PPP), palmoplantar psoriasis (palmPP), and dyshidrotic palmoplantar eczema (DPE). Their findings highlight shared upregulation of proinflammatory cytokines, chemokines, and T-cell–associated genes. Research in this field often lacks rigorous inclusion criteria, such as consistent diagnostic and sampling standards, making studies such as theirs—which has high reproducibility—extremely valuable for advancing global clinical trials. We sincerely commend their work. In addition to identifying shared molecular characteristics, the study elucidated unique features of each disease, such as enriched neutrophil processes in PPP (and, to a lesser extent, in palmPP) and lipid antigen processing in DPE. ^1^ These findings further clarify the histological and pathophysiological differences between PPP and DPE, which are of particular interest to us.

PPP is a persistent pustular skin condition that primarily affects the palms and soles. It is marked by the presence of erythema, pustules, and irregular peeling of the skin with or without psoriasis vulgaris. ^2^ In Japan, PPP rarely co-occurs with psoriasis, whereas in Western countries, it is frequently associated with psoriasis. Our group has proposed subdividing PPP into type A (rarely associated with psoriasis) and type B (frequently associated with psoriasis). ^2^ Type A PPP is distinguished by the clinical feature of intraepidermal vesicles preceding pustule formation. ^2^ We recently demonstrated that interleukin (IL)-1 present in eccrine sweat could infiltrate the epidermis via the acrosyringium, impairing E-cadherin expression on keratinocytes and contributing to the development of intraepidermal vesicles in type A PPP. ^3^ Conversely, the mechanisms underlying intraepidermal vesicle formation in DPE remain poorly understood. Spongiosis, a hallmark of eczema, is associated with hyaluronan production and reduced E-cadherin expression, driven by IL-4, IL-13, and interferon-γ (IFN-γ)-stimulated keratinocytes. ^4^ Notably, we previously observed that keratinocytes around PPP-associated vesicles lack hyaluronan expression, unlike those in DPE. ^5^ These findings strongly suggest that the mechanisms of intraepidermal vesicle formation differ between PPP and DPE.

Straalen et al. confirmed and redefined clinical diagnoses of PPP and DPE using biopsy samples, diagnosing PPP based on “intraepidermal vesicles” without spongiosis and with potential microabscesses at the edges. ^1, 6^ These criteria align with type A PPP, increasing our interest in their study. The authors reported higher expression of IL-13 and IFN-γ in DPE than in PPP, ^1^ which aligns with our understanding and represents an intriguing confirmation of these findings.

To elucidate further the pathophysiology of PPP and DPE, we invite the authors to share their opinions and any detailed findings regarding IL-4 expression, hyaluronan synthases 1 to 3, and intercellular adhesion factors of epidermal keratinocytes, such as cadherins and desmogleins.

## Ethics and consent

Ethical approval and consent were not required.

## Data Availability

No data are associated with this article.

## References

[1] Straalen KR van Kirma J Yee CM : Disease heterogeneity and molecular classification of inflammatory palmoplantar diseases. *J. Allergy Clin. Immunol.* 2024;154(5):1204–1215.e9. 10.1016/j.jaci.2024.07.017 39089334

[2] Murakami M Terui T : Palmoplantar pustulosis: Current understanding of disease definition and pathomechanism. *J. Dermatol. Sci.* 2020;98(1):13–19. 10.1016/j.jdermsci.2020.03.003 32201085

[3] Yatsuzuka K Kawakami R Niko Y : A fluorescence imaging technique suggests that sweat leakage in the epidermis contributes to the pathomechanism of palmoplantar pustulosis. *Sci. Rep.* 2024;14(1):378. 10.1038/s41598-023-50875-x 38172327 PMC10764317

[4] Ohtani T Memezawa A Okuyama R : Increased hyaluronan production and decreased E-cadherin expression by cytokine-stimulated keratinocytes lead to spongiosis formation. *J. Invest. Dermatol.* 2009;129(6):1412–1420. 10.1038/jid.2008.394 19122650

[5] Murakami M Muto J Masuda-Kuroki K : Pompholyx vesicles contain small clusters of cells with high levels of hyaluronate resembling the pustulovesicles of palmoplantar pustulosis. *Br. J. Dermatol.* 2019;181(6):1325–1327. 10.1111/bjd.18261 31254390

[6] Masuda-Kuroki K Murakami M Kishibe M : Diagnostic histopathological features distinguishing palmoplantar pustulosis from pompholyx. *J. Dermatol.* 2019;46(5):399–408. 10.1111/1346-8138.14850 30919463

