# Supplementary material for: Comments about the comparative bulk RNA sequencing between palmoplantar pustulosis and dyshidrotic palmoplantar eczema
Source: F1000Res. 2024 Dec 6;13:1494. [Version 1] doi: 10.12688/f1000research.159565.1 (PMC11729159; doi:10.12688/f1000research.159565.1)
Supplement: Peer review history

# Reviewer report: Reviewer response for version 1

**DOI:** 10.5256/f1000research.175314.r352003

**Published:** 2025-01-13

**Authors:** Sueli Carneiro

**Affiliations:** 1 Federal University of Rio de Janeiro, RJ, Brazil

The authors agree with the findings of Straalen et al.  and confirm that the redefinition of the clinical diagnoses of PPP and DPE were of great clinical and histopathological value. The authors further state that such findings, criteria and definitions align with their type A PPP criteria, including in relation to greater expression of IL-13 and IFN-γ in DPE than in PPP.

 They close the communication by inviting authors to share their opinions and any other detailed findings on the expression of interleukins and cell aggregation factors.

Are arguments sufficiently supported by evidence from the published literature or by new data and results?

Yes

Is the conclusion balanced and justified on the basis of the presented arguments?

Yes

Is the rationale for commenting on the previous publication clearly described?

Yes

Are any opinions stated well-argued, clear and cogent?

Yes

Reviewer Expertise:

Dermatology, Rheumatology and Immunodermatology and Immunorheumatology

I confirm that I have read this submission and believe that I have an appropriate level of expertise to confirm that it is of an acceptable scientific standard.

# Reviewer report: Reviewer response for version 1

**DOI:** 10.5256/f1000research.175314.r352002

**Published:** 2025-01-07

**Authors:** Yulong Sun, Huijuan Li

**Affiliations:** 1 Northwestern Polytechnical University, Xi'an, Shaanxi, China; 2 School of Electrical Engineering, Shaanxi Polytechnic Institute, Xianyang, Shaanxi, China

The advent of high-throughput omics technologies, particularly bulk transcriptome sequencing, has revolutionized our understanding of complex diseases over the past two decades. This innovative approach has been crucial for elucidating disorders with overlapping clinical presentations, such as palmoplantar pustulosis (PPP), non-pustular palmoplantar psoriasis (palmPP), and wet palmoplantar eczema (DPE). In this context, the work of Straalen et al. offers valuable insights into the transcriptomic profiles of these closely related conditions. Their study includes a comprehensive analysis of disease samples, comprising lesion samples from PPP (n = 533), palmPP (n = 555), and DPE (n = 528), alongside skin samples from 10 healthy individuals and 5 healthy non-acral sites. This systematic mapping enhances our understanding of these three diseases and highlights the distinctive features of their immune microenvironments ^1^.

 Kazuki Yatsuzuka et al. have provided a comprehensive summary of the work by Straalen et al., effectively analyzing the clinical characteristics and challenges associated with diagnosing and treating three specified skin diseases ^2^. They articulate the clinical significance of this research, emphasizing its relevance for improving diagnostic and therapeutic strategies in this field. The authors recommend a more nuanced molecular classification of Palmoplantar Psoriasis (PPP), particularly considering the frequent co-occurrence of psoriasis, which may enhance awareness and understanding of this condition. Furthermore, the authors elucidate the differing mechanisms underlying intraepidermal vesicle formation in each disorder by examining the pathophysiological and histological distinctions between PPP and Dyshidrotic Eczema (DPE). This insight offers a valuable foundation for accurately diagnosing and treating these often-confused conditions. Overall, this work has significant implications for advancing our understanding of dermatological research and clinical practice.

Are arguments sufficiently supported by evidence from the published literature or by new data and results?

Yes

Is the conclusion balanced and justified on the basis of the presented arguments?

Yes

Is the rationale for commenting on the previous publication clearly described?

Yes

Are any opinions stated well-argued, clear and cogent?

Yes

Reviewer Expertise:

psoriasis

We confirm that we have read this submission and believe that we have an appropriate level of expertise to confirm that it is of an acceptable scientific standard.

## References

[1] : Disease heterogeneity and molecular classification of inflammatory palmoplantar diseases. *J Allergy Clin Immunol* .2024;154(5) : 10.1016/j.jaci.2024.07.017 1204-1215.e9 10.1016/j.jaci.2024.07.017 39089334
